# Intrinsically Healable and Photoresponsive Electrospun Fabrics: Integrating PVDF-HFP, TPU, and Azobenzene Ionic Liquids

**DOI:** 10.1021/acsami.4c17199

**Published:** 2024-12-23

**Authors:** Chun-Chi Chang, Lin-Ruei Lee, Sheng Zheng, Tse-Yu Lo, Chia-Wei Chang, Chia-Ti Wu, Tsung-Hung Tsai, Huan-Ru Chen, Yi-Fan Chen, Ming-Hsuan Chang, Jiun-Tai Chen

**Affiliations:** † Department of Applied Chemistry, National Yang Ming Chiao Tung University, Hsinchu, Taiwan 300093; ‡ Center for Emergent Functional Matter Science, National Yang Ming Chiao Tung University, Hsinchu, Taiwan 300093

**Keywords:** electrospinning, intrinsic self-healing, ionic liquids, photoresponsive, azobenzene

## Abstract

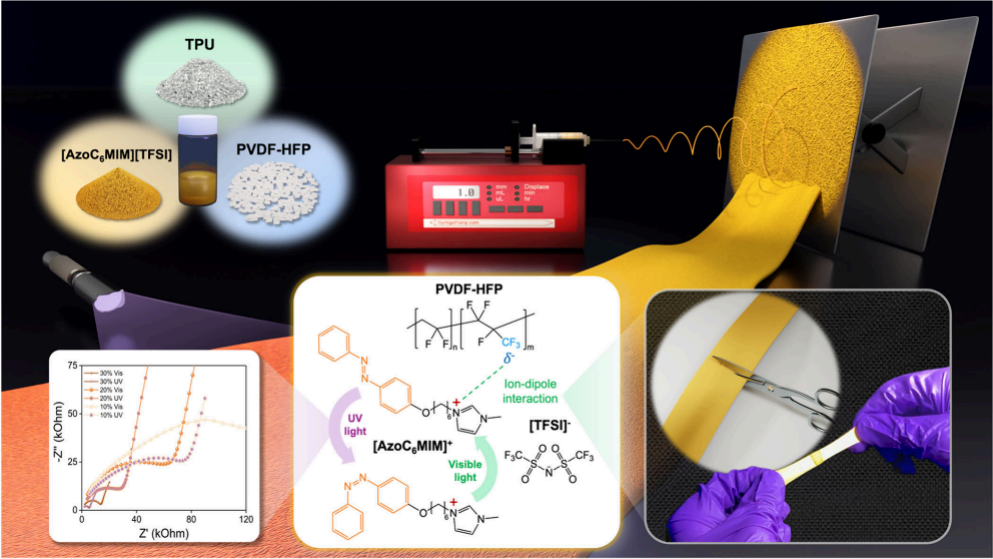

In recent years, the integration of multifunctional properties into electrospun fabrics has garnered significant attention for applications in wearable devices and smart textiles. A major challenge lies in achieving a balance among intermolecular interactions, structural stability, and responsiveness to external stimuli. In this study, we address this challenge by developing intrinsically healable and photoresponsive electrospun fabrics composed of poly(vinylidene fluoride-*co*-hexafluoropropylene) (PVDF-HFP), thermoplastic polyurethane (TPU), and an azobenzene-based ionic liquid ([AzoC_6_MIM][TFSI]). The interactions between PVDF-HFP and [AzoC_6_MIM][TFSI] enable intrinsic self-healing and light-induced responsiveness, while the incorporation of TPU prevents fiber fusion during electrospinning, maintaining structural integrity and porosity. Our results demonstrate that these fabrics can recover up to 97% of their original mechanical properties after self-healing and exhibit reversible changes in electrical conductivity under UV and visible lights. This versatile approach paves the way for the incorporation of high concentrations of functional ionic liquids into electrospun fabrics, enabling the development of multifunctional textiles with potential applications in self-healing wearable devices and advanced sensors.

## Introduction

Electrospinning has been developed as an effective and efficient technique to fabricate polymer micro- or nanofibers.^1−4^ In recent years, electrospun polymer fibers with functionalities have captured more attention because of their diverse applications, such as self-healing materials, wearable electronics, energy system, and drug delivery.^5−7^ These fibers provide unique advantages including tunable properties and morphologies, high surface area-to-volume ratios, and the ability to incorporate various functional materials.^8−10^ One of the methods to fabricate multifunctional electrospun polymer fabrics is by incorporating functional tunable ionic liquids into polymers for electrospinning.^11,12^ The versatility of ionic liquids can lead to intricate interactions, such as Coulomb forces, van der Waals forces, and hydrogen bonding.^13−17^ Therefore, when blended with polymers, the interactions of ionic liquids can affect the properties of electrospinning solutions, such as viscosity, density, solubility, conductivity, and hydrophobicity; as a result, electrospun fibers with various morphologies can be achieved.^18^ Moreover, there are additional advantages using ionic liquids, such as low vapor pressure and high thermal stability to prevent materials from evaporation and degradation.^19^ On the other hand, ionic liquids can be tailored to exhibit various stimuli-responsive properties by modifying the functional groups of their cations, anions, or both.^20,21^ After being incorporated with functionalized ionic liquids, materials can possess responsiveness to different kinds of stimuli,^22,23^ such as light,^24−27^ temperature,^28−30^ pH value,^31,32^ and gas.^33^ Thus, multifunctional polymer fabrics can be fabricated by combining stimuli-responsive ionic liquids and the electrospinning process.

For electrospun fibers, the self-healing property is another important functionality because it leads to potential applications in environmental reusability and sustainability.^34−37^ In general, intrinsic self-healing materials can leverage the inherent properties to achieve repetitive healing capability without external intervention;^38^ by comparison, extrinsic self-healing materials often heal only once by using additional healing agents.^39−42^ To fabricate intrinsic self-healing materials, various interactions can be utilized, such as ion-dipole interactions,^43^ hydrogen bonding,^44^ and metal–ligand interaction.^45^ The ion-dipole interactions present between ionic liquids and polymers with strong dipole moments can endow fabrics with self-healing abilities.^46,47^ The addition of ionic liquids to polymers, however, could cause fusing between fibers because of excessively strong interactions; therefore, the fusing process results in collapsed and melted morphologies with small or no pores, affecting breathability and effective surface areas of fabrics. Moreover, after adding ionic liquids, the increased conductivities of electrospinning solutions also make it more challenging to accumulate charges for the electrospinning process. Previously, we investigated the fabrication of self-healing electrospun fibers by mitigating the problems of fibers fusion through employing high voltages (up to 30 keV), altering environment conditions, adjusting solution formulations (such as viscosity, concentration <10 wt %, or polymer crystallinity), and using different collection methods such as wet electrospinning.^47,48^ These approaches, however, may compromise the functionalities of the materials or increase the complexity and cost of the processing.

To address the above issues, in this study, we develop photoresponsive electrospun polymer fabrics with healing ability by adding photoresponsive ionic liquids, polymers with strong dipole moments, and thermoplastic elastomers. An azobenzene-based ionic liquid ([AzoC_6_MIM][TFSI]), poly(vinylidene fluoride-*co*-hexafluoropropylene) (PVDF-HFP), and a thermoplastic elastomer (thermoplastic polyurethane, TPU) are mixed as the electrospinning solution to fabricate photoresponsive polymer fabrics. TPU is used to avoid the fusing of the fibers and to maintain and control the structural stability of the electrospun fabrics, ensuring adequate breathability and interfiber porosity. The presence of TPU in the fabrics enables the concentration of ionic liquids to reach over 20 wt %. Moreover, the interactions between PVDF-HFP and [AzoC_6_MIM][TFSI] are utilized to achieve the self-healing properties. With the photoresponsive azobenzene moiety of the ionic liquids, the fabrics can be reversibly controlled by UV and visible lights through isomerization of azobenzene. Besides, PVDF-HFP and TPU provide the lightness and mechanical strength of the fabrics, while incorporated [AzoC_6_MIM][TFSI] offers thermal stability and conductivity. This approach opens new possibilities for incorporating high concentrations of functional ionic liquids into electrospun fabrics; the development of multifunctional fabrics should have potential applications in different areas such as light sensors, multifunctional clothing, and wearable devices.

## Results and Discussion

### Design of the Intrinsically Healable and Photoresponsive Electrospun Fabrics

**Figure 1:**
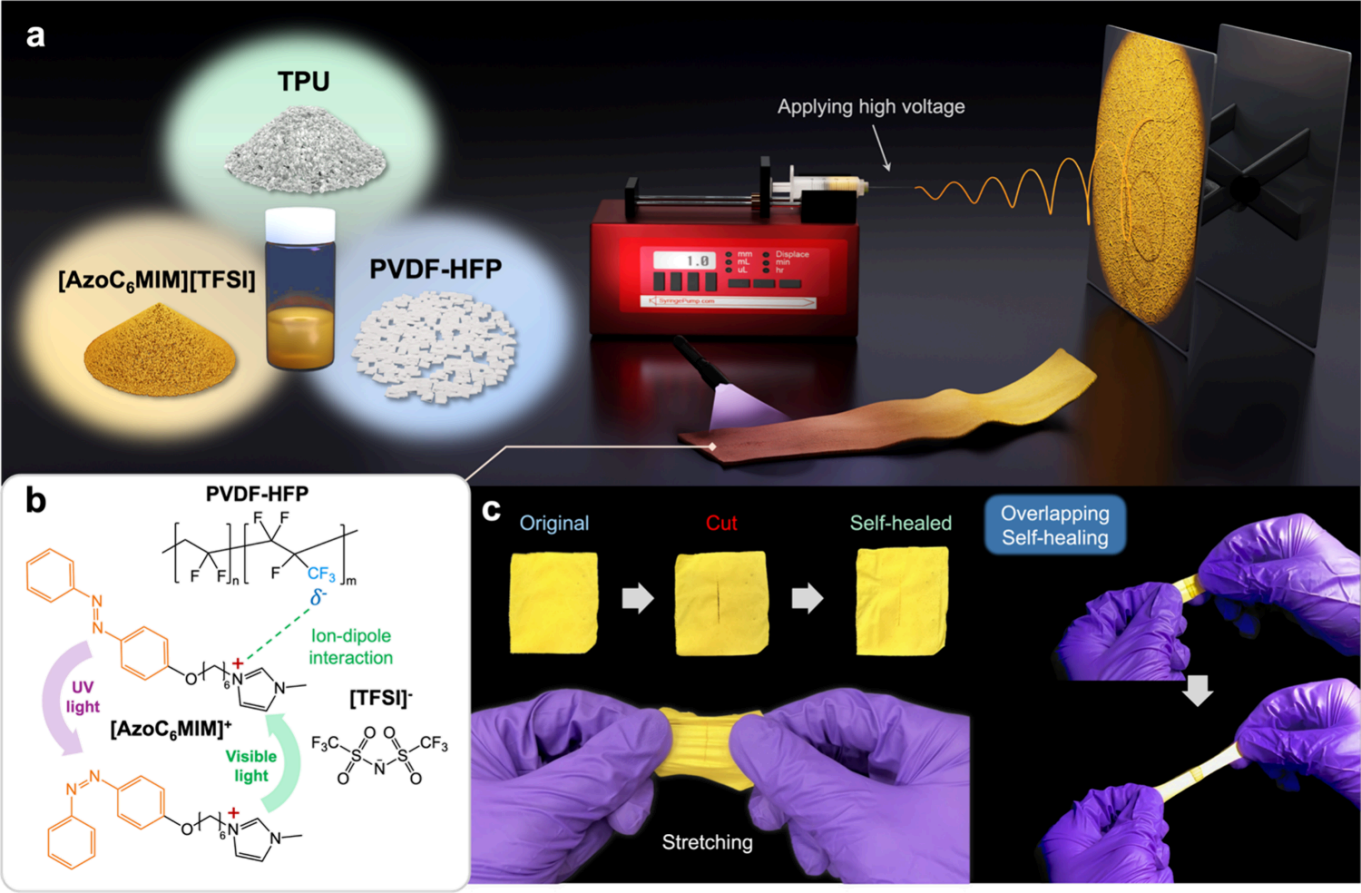
(a) Schematic illustration to prepare the electrospun fabrics. (b) Schematic illustration of the interactions between [AzoC_6_MIM][TFSI] and PVDF-HFP and the photoresponsiveness of [AzoC_6_MIM][TFSI]. (c) Photos of original and posthealed fabrics.

As shown in Figure 1a, azobenzene ionic liquid ([AzoC_6_MIM][TFSI]), PVDF-HFP, and TPU are blended to prepare a mixed solution. In addition, the electrospinning method with a high voltage is used to spray the polymer solution and form microfibers. In the electrospinning device, a high voltage is applied to the solution from the needle of the syringe. The electrospun fabrics can be collected with aluminum foils. When irradiated with UV light, the colors of the fabrics change from yellow to orange because of the isomerization of azobenzene.

As shown in Figure 1b, the isomerization of azobenzene (*cis* and *trans*) can be controlled by UV and visible lights. Similarly, azobenzene-based ionic liquids also exhibit photoresponsive properties. Photoisomerization alters the structures of azobenzene ionic liquids, which influences the charge behaviors and aggregating tendencies of compounds.^27,49^ Therefore, light irradiation can induce changes in the conductivities. PVDF-HFP is a copolymer composed of VDF and HFP units, as shown in Figure 1b. In the polymer, VDF is less polar but more crystalline, and HFP is more polar but amorphous, which contributes to the main dipole characteristics of the polymer. Therefore, PVDF-HFP with a high HFP ratio can enhance dipole moment interactions with azobenzene-based ionic liquids. In this study, PVDF-HFP is used with 55 mol % VDF. The copolymerization with HFP disrupts the original crystalline α-phase of PVDF, promoting more β-phase alignments. Besides, the high-voltage electrospinning process also induces more β-phase alignments in PVDF-HFP. These β-phase alignments of PVDF-HFP result in stronger dipole moments. In this study, the dipole moments of PVDF-HFP interact with the ionic charges of azobenzene-based ionic liquids. The schematic illustration of the ion-dipole interactions is also shown in Figure 1b. The attractive interaction can be used as an intrinsic self-healing ability of fibers. Microfibers are fabricated by a mixed solution including the azobenzene-based ionic liquid ([AzoC_6_MIM][TFSI]), PVDF-HFP, and TPU using the electrospinning method. Figure 1c depicts the self-healing applications of electrospun fabrics. When posthealed fabrics are stretched, the approached and overlapped regions do not exhibit any fracture, which verifies the intrinsic self-healing ability of the fabrics.

### Synthesis and Characterization of [AzoC_6_MIM][TFSI]

**Figure 2:**
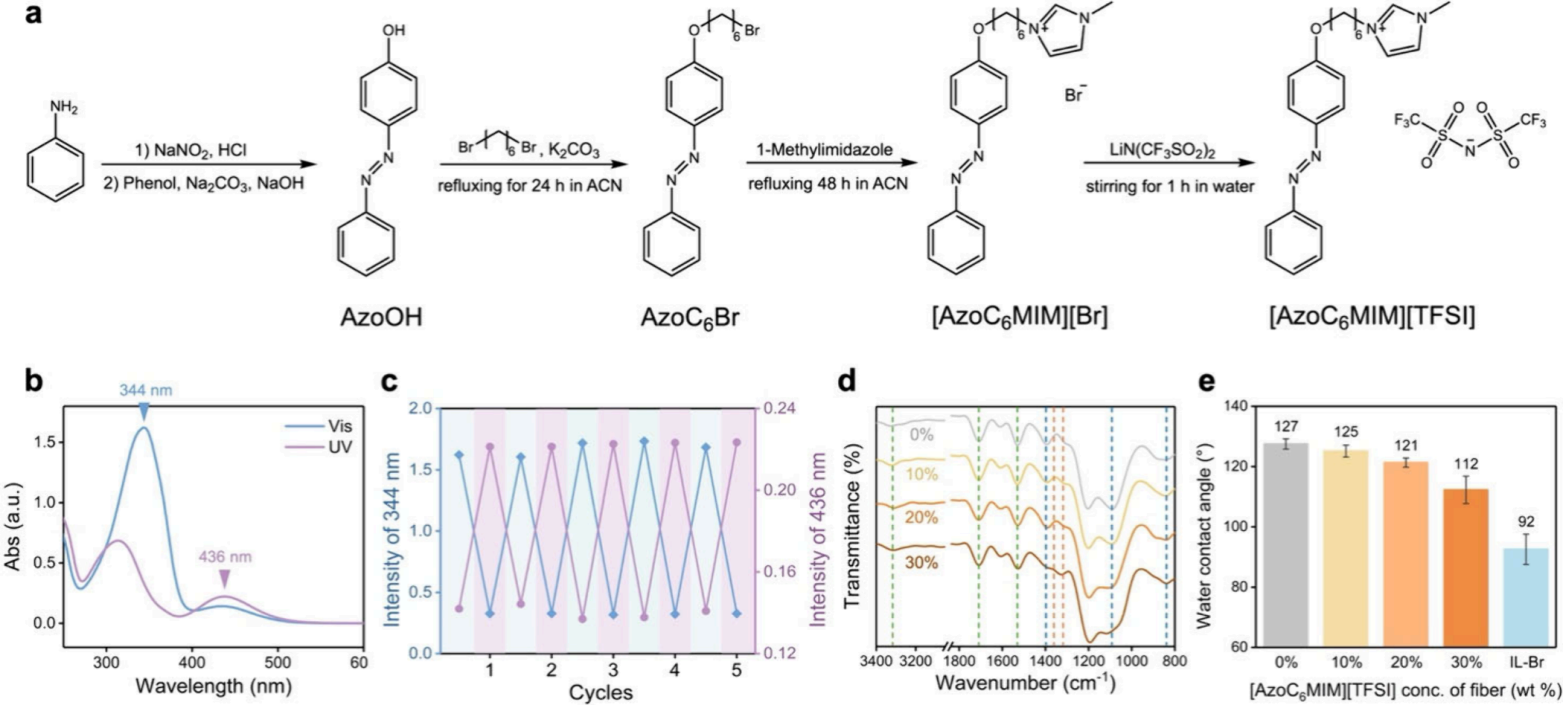
(a) Synthetic route of [AzoC_6_MIM][TFSI]. (b) UV–vis absorption spectra of 0.2 mM *trans*- and *cis*-[AzoC_6_MIM][TFSI] in an ethanol solution. (c) Reversible test of [AzoC_6_MIM][TFSI] isomerization under alternating UV and visible light irradiations. The irradiation times of UV and visible light are 60 s and 3 min, respectively. (d) FTIR spectra of fabrics with different concentrations of [AzoC_6_MIM][TFSI]. (e) Plot of water contact angles of fabrics with different concentrations of [AzoC_6_MIM][TFSI] and [AzoC_6_MIM][Br].

As illustrated in Figure 2a, an azobenzene-based ionic liquid ([AzoC_6_MIM][TFSI]) is synthesized through an alkylation reaction using 1,6-dibromohexane and a subsequent quaternization reaction with 1-methylimidazole to form the ionic liquid. Finally, the desired product, [AzoC_6_MIM][TFSI], is obtained by an anion exchange reaction to replace [Br^–^] with [TFSI^–^]. The corresponding ^1^H and ^19^F NMR spectra of [AzoC_6_MIM][TFSI] are shown in Figure S1. The peak at −80.30 ppm in the ^19^F NMR spectrum suggests that [AzoC_6_MIM][TFSI] has been successfully synthesized. The azobenzene moieties in the ionic liquids endow the compounds with photoresponsiveness. When the imidazole groups are grafted to the azobenzene molecules, forming ionic liquids, they would enhance the thermal stability and conductivity. Besides, the bromide anions are replaced with [TFSI]^−^ to improve conductivity, thermal stability, and hydrophobicity.^19,50−52^

Figure 2b displays the UV–vis absorption spectra of the [AzoC_6_MIM][TFSI] solution under two light-irradiating conditions. Under visible light, the maximum absorption wavelength is 344 nm, indicating the π–π* transition of the *trans*-[AzoC_6_MIM][TFSI]. Upon UV light irradiation, the absorption intensity at 344 nm decreases, and the wavelength exhibits blue shift. Moreover, there is a slight increase in intensity at 436 nm, indicating the n−π* transition of the *cis* isomer. Alternating irradiations between two wavelengths (UV and visible lights) are used to conduct a reversibility test. As shown in Figure 2c, reversible intensity changes at wavelengths of 344 and 436 nm are observed, which demonstrates the reversibility of [AzoC_6_MIM][TFSI] isomerization (*cis* and *trans*).

As shown in Figure 2d, FTIR spectra are employed to characterize the fabrics. The wavenumber at 840 cm^–1^ corresponds to the C–F stretching vibration of the amorphous phase of PVDF-HFP. The peak observed at 1710 cm^–1^ is attributed to the C=O stretching vibration of TPU. The incorporation of [AzoC_6_MIM][TFSI] into fabrics is confirmed by the presence of a peak at 1352 cm^–1^, which corresponds to the N=N stretching vibration of the azobenzene moiety. Detailed information about the FTIR spectra is provided in Table S1. As shown in Figure 2e, the hydrophobicity of the fabrics is attributed to the high fluorine content in PVDF-HFP. A slight decrease in water contact angles is observed with increasing concentrations of [AzoC_6_MIM][TFSI]. In contrast, fabrics containing [AzoC_6_MIM][Br] exhibit a more pronounced decrease in contact angles because of their hydrophilic properties. The anion exchange from [AzoC_6_MIM][Br] to [AzoC_6_MIM][TFSI] decreases the water solubility, thereby enhancing the waterproof characteristics of the fabrics. Additionally, the energy-dispersive X-ray spectroscopy (EDS) data of the fabrics are also shown in Figure S2, demonstrating that phase separation does not occur in the fibers. Moreover, the TGA curves of the fabrics and polymers are presented in Figure S3 to show the thermal stabilities of the fabrics.

### Effect of TPU and [AzoC_6_MIM][TFSI] on Fiber Morphologies

**Figure 3:**
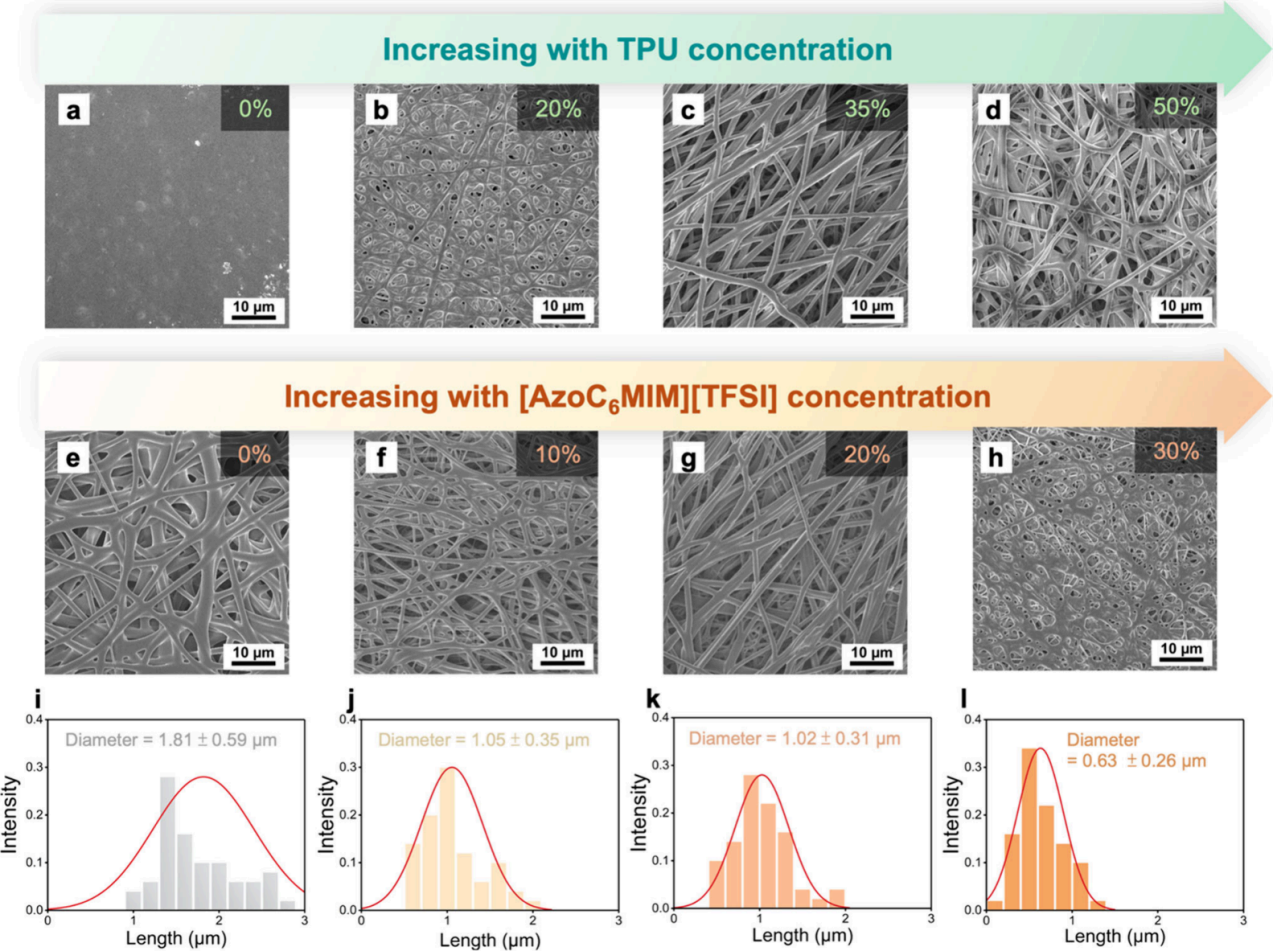
(a–d) SEM images of fabrics with increasing contents of TPU in the fixed concentration (20 wt %) of [AzoC_6_MIM][TFSI]: (a) 0, (b) 20, (c) 35, and (d) 50 wt % of TPU. (e–h) SEM images of fabrics with increasing contents of [AzoC_6_MIM][TFSI]: (e) 0, (f) 10, (g) 20, and (h) 30 wt % of [AzoC_6_MIM][TFSI]. The ratio of PVDF-HFP:TPU = 5:4. (i–l) Corresponding diameter distributions of the fabrics shown in (e–h).

In this study, microfibers are fabricated by a mixed solution including the azobenzene-based ionic liquid ([AzoC_6_MIM][TFSI]), PVDF-HFP, and TPU using the electrospinning method. In this work, only [AzoC_6_MIM][TFSI] instead of [AzoC_6_MIM][Br] is used in the electrospun fabrics because of the better conductivity and hydrophobicity of [AzoC_6_MIM][TFSI]. TPU is incorporated into the polymer solutions to prevent fibrous structures from fusing. The SEM images of fibers with TPU of different concentrations are shown in Figure 3a–d. The morphologies of fibers change significantly with variation of the TPU concentrations. Initially, the fabric without TPU forms a nearly film-like structure instead of a porous fibrous structure (Figure 3a). When TPU is incorporated, the fibrous structure is stably formed because TPU increases the viscosity of the solutions, which prevents the solutions from spraying out as an unstable jet (Figure S4). Therefore, as the concentration of TPU increases (Figure 3a–d), fiber fusion is avoided during the electrospinning process, and more stable and robust fibrous structures are formed, resulting in larger fabric porosity. However, at higher TPU concentrations, the electrospun fabrics exhibit shrinkage due to the intrinsic elastic properties of TPU. This shrinkage causes the fibers to curl, leading to reduced fiber gaps in the structure, as shown in Figure 3d. Furthermore, the addition of TPU in solutions allows the fabrication of fabrics at a lower voltage (20 keV) without the need to reduce the amounts of ionic liquids. Additionally, TPU intrinsically exhibits structural stability and good dispersibility, allowing TPU to blend with PVDF-HFP and azobenzene ionic liquids uniformly. The uniform blending reduces the probabilities of phase separations and intense interactions between the PVDF-HFP and the ionic liquids.

Regarding the influence of [AzoC_6_MIM][TFSI] on the morphologies of fabrics, it is observed that the fibers are fused with the increasing concentrations of [AzoC_6_MIM][TFSI], as shown in Figure 3e–h and Figure S5. Higher concentrations of ionic liquids may cause better conductivities and stronger interactions with PVDF-HFP in the fabrics. Excessive amounts of ionic liquids (greater than 30 wt % of [AzoC_6_MIM][TFSI]), however, cause fibers to fuse with adjacent ones, which reduces porosity and forms an unstable structure. Moreover, the corresponding diameter distributions are shown in Figure 3i–j. It is implied that as the concentration of [AzoC_6_MIM][TFSI] increases from 0 to 20 wt %, the diameters of the fibers decrease with narrower distributions. At higher concentrations of [AzoC_6_MIM][TFSI] (30 wt % and higher), although the fiber diameters further decrease, the fibers partially melt, forming film-like regions. Moreover, when the concentration reaches 40 wt %, films instead of fabrics are mostly collected. The influence of TPU and [AzoC_6_MIM][TFSI] on the heights and morphologies is also demonstrated by the AFM images, as shown in Figure S6.

### Mechanical Properties and Self-Healing Behavior

**Figure 4:**
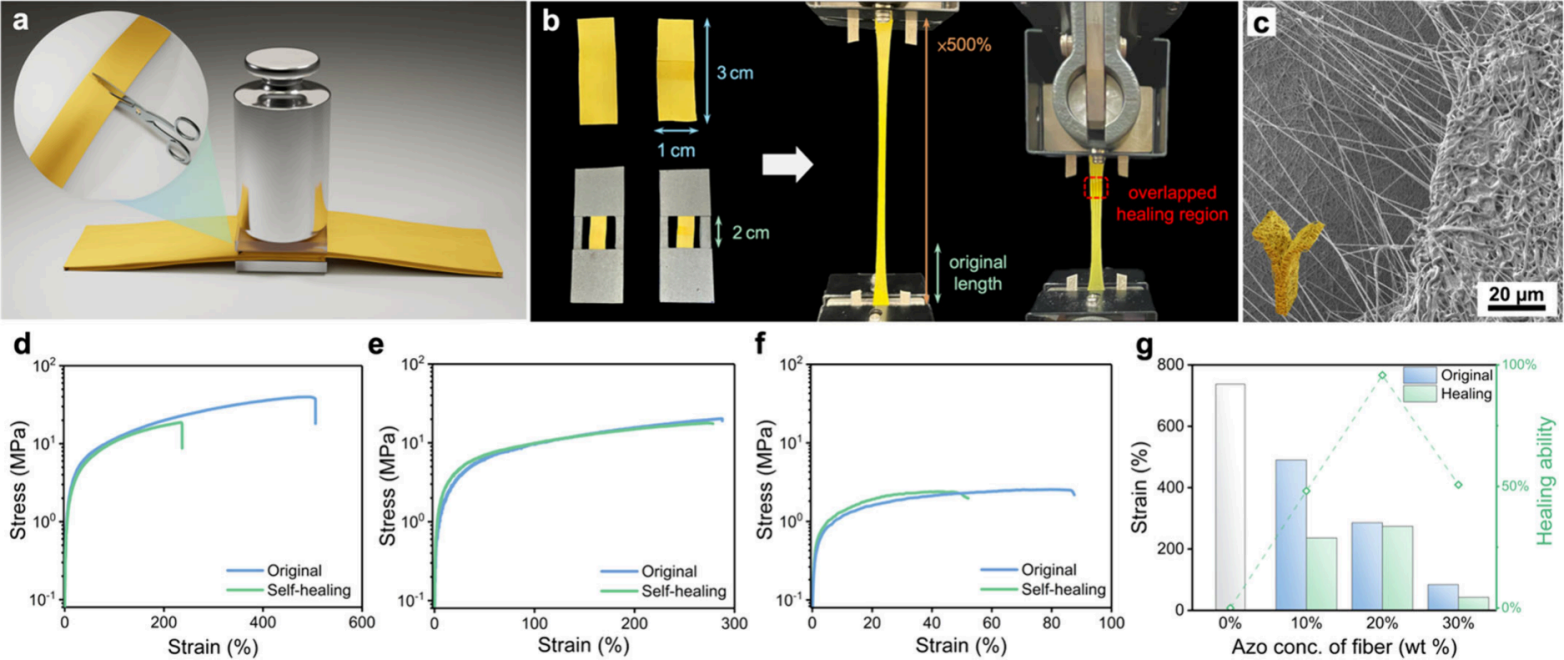
(a) Schematic illustration of the cutting and pressing process for a healing test. (b) Real image of a tensile test. The stretched areas of the fabrics are fixed at 0.5 cm^2^. (c) Top-view SEM image of a T-shape self-healing fabric. (d–f) Stress–strain curves of fabrics with different concentrations of [AzoC_6_MIM][TFSI] obtained from a tensile test: (d) 10, (e) 20, and (f) 30 wt % of [AzoC_6_MIM][TFSI]. (g) Plot of the strain and healing abilities of fabrics before and after self-healing.

The intrinsically healable electrospun fabrics should be capable of healing without using any additives. In this study, ion-dipole interactions between PVDF-HFP and [AzoC_6_MIM][TFSI] are leveraged to achieve intrinsic self-healing. Intrinsic healing typically requires materials with a low glass transition temperature (*T*_g_) to facilitate chain diffusion and rearrangements. As shown in the DSC spectra (Figure S7), the *T*_g_ values of tested fabrics with or without adding ionic liquids are all below room temperature (from −20 to −40 °C), which suggests the intrinsic healing capability of the fibers. Figure 4a shows a schematic illustration of the healing process. To evaluate self-healing, the fabric is cut with a scissor, and the two cut pieces are overlapped, followed by covering glass slides. A weight of 1 kg is then placed on the sample to apply the pressure. The setup is used to simulate the condition of a fabric with a fracture. Besides, as shown in Figure 4b, the self-healing abilities and mechanical properties are determined using a tensile test. Moreover, the T-shape healing process is utilized to evaluate the actual fabric structure. As shown in Figure 4c, the top-view SEM image shows that the fibers clearly cross-link to each other, which forms intertwined networks between two fabrics. The observation confirms the healing ability through structural entanglements of fibers.

The mechanical properties of fabrics are also compared before and after healing. Figure 4d–f displays the mechanical properties of fabrics with varying concentrations of [AzoC_6_MIM][TFSI] from 0 to 30 wt %. The remaining portion of the polymer matrix (70–100 wt %) consists of PVDF-HFP and TPU in a fixed ratio of 5:4. In the tensile test, different concentrations of [AzoC_6_MIM][TFSI] also refer to different concentrations of PVDF-HFP and TPU complementarily. The results show that the fabrics exhibit notable stretchability and tensile strength. The tensile strains of the fabrics without fractures reach 490, 286, and 84% in the samples containing 10, 20, and 30 wt % of [AzoC_6_MIM][TFSI], respectively. Moreover, it is observed that the corresponding tensile strains of the posthealed fabrics reach 236, 274, and 50%. As a result, the posthealed fabrics with 20 wt % of [AzoC_6_MIM][TFSI] almost restore their original mechanical properties. As shown in Figure 4g, the trends of tensile stresses and strains indicate that the mechanical properties of the fabrics are related to the concentrations of polymers (PVDF-HFP and TPU), which can be attributed to the intrinsic properties of TPU. However, a high TPU ratio (50 wt %) results in weak fiber interactions, potentially diminishing the self-healing capabilities, as shown in Figure S8. It is determined that a TPU concentration from 30 to 40 wt % strikes an optimal balance between the structural stability and the functionality of ion-dipole interactions.

It can be noted that at higher ionic liquid concentrations (lower polymer concentrations), the fabrics become more fragile and easily break under tensile forces. The results are due to less amounts of polymers and the excessive ionic liquids, which disrupt the polymer matrix. It is suggested that the concentrations of the ionic liquids can influence the fibrous performance in terms of both mechanical properties and self-healing abilities. Furthermore, to obtain an accurate self-healing ability, the healing ratios are also calculated from the results of the tensile test. The analyses reveal that the healing ability is not only dependent on the concentration of the ionic liquids but also related to the concentration of PVDF-HFP. When more [AzoC_6_MIM][TFSI] is added into the fabrics, the amounts of PVDF-HFP in the fabrics simultaneously decrease. This reduction in PVDF-HFP (a strongly dipolar polymer) influences the essential ion-dipole interactions required for effective self-healing. Consequently, it is observed that the fabric with 20 wt % [AzoC_6_MIM][TFSI] reaches the optimal healing ratio of 97%. In contrast, the fabric with a 30 wt % of [AzoC_6_MIM][TFSI] only attains a healing ratio of 50%, along with an obvious loss in mechanical properties. As shown in Figure S9, the cyclic tensile test performed 10 times at a fixed strain reveals the differences in mechanical stresses of fabrics with varying concentrations of [AzoC_6_MIM][TFSI]. These results highlight the importance of balancing the concentrations of ionic liquids and PVDF-HFP to enhance the self-healing ability of the fabrics.

### Photoresponsive Electrical Properties

**Figure 5:**
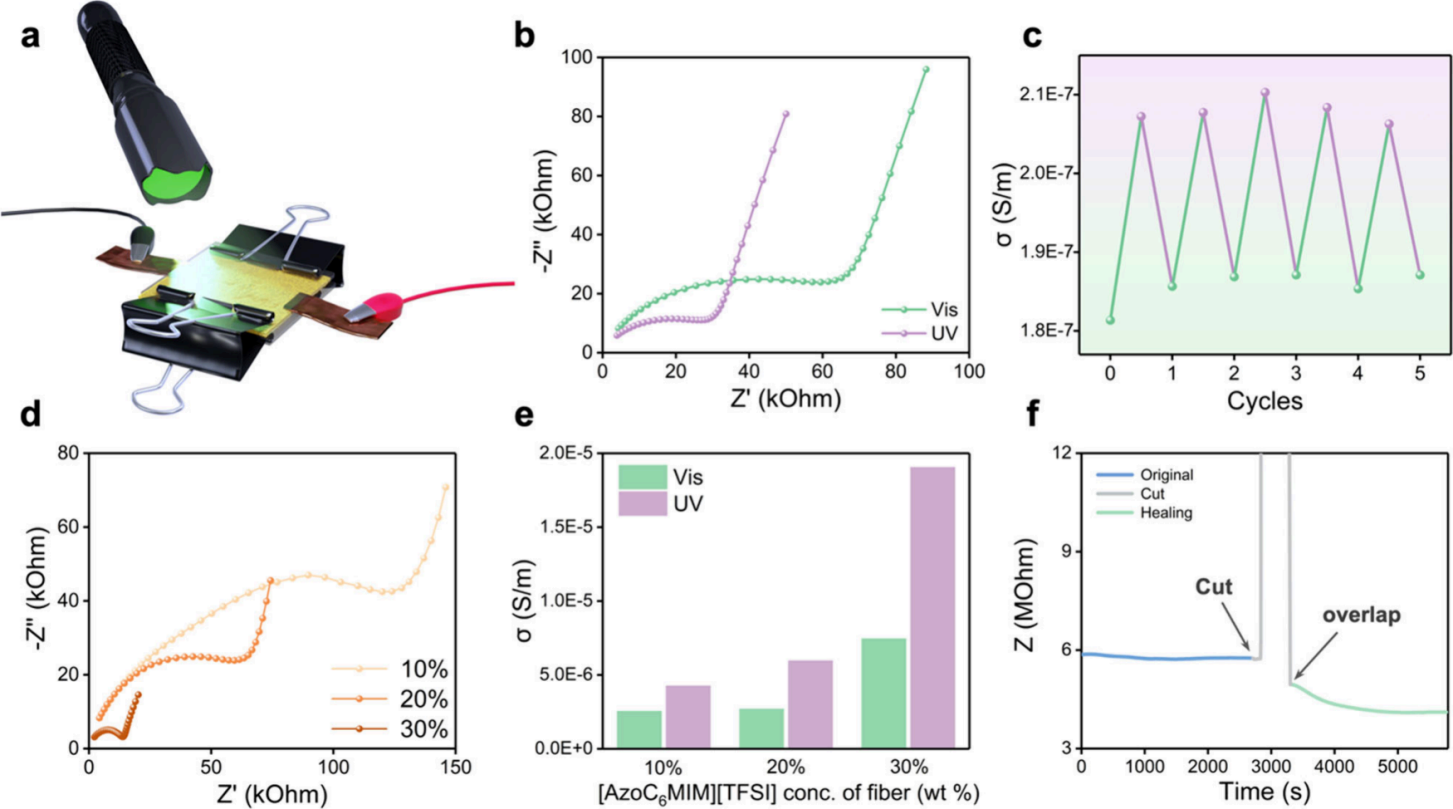
(a) Schematic illustration of a device to measure the electrical property of a fabric. (b) Nyquist plots and (c) reversible conductivities using a fabric containing 20 wt % [AzoC_6_MIM][TFSI] under UV and visible light irradiations. (d) Nyquist plots of fabrics with 10, 20, and 30 wt % of [AzoC_6_MIM][TFSI]. (e) Plots of conductivities of fabrics with different concentrations under UV and visible light irradiations. (f) Plot of time-resolved impedance during the self-healing process.

The fabrics not only feature enhanced conductivities because of the ionic liquids but also exhibit photoresponsive controllability by UV and visible lights due to the presence of [AzoC_6_MIM][TFSI]. Figure 5a shows the device for measuring the electrical properties. The electrical properties of the fabrics are measured with electrochemical impedance spectroscopy (EIS). Figure 5b shows the Nyquist plots of the fabrics under visible and UV light irradiations. The results demonstrate that the impedance decreases after the sample is exposed to UV light irradiation. It is speculated that the increased conductivity is due to the isomerization to *cis*-[AzoC_6_MIM][TFSI]. After exposure to UV light, the azobenzene part of [AzoC_6_MIM][TFSI] transforms to less bulky *cis*-[AzoC_6_MIM][TFSI], which highlights the influence of higher conductive imidazolium cations. Moreover, the mobility of *cis*-[AzoC_6_MIM][TFSI] is higher than those of *trans*-[AzoC_6_MIM][TFSI] because of reduced amphiphilic self-aggregated tendency.^27^ The reversibility of the conductivity is demonstrated in Figure 5c over five cycles. The conductivities remain in the range of 1.8 × 10^–7^ to 1.9 × 10^–7^ under visible light irradiation; similarly, the conductivities remain in the range of 2.0 × 10^–7^ to 2.2 × 10^–7^ under UV light irradiation. These results suggest that the conductivities of the fabrics can be precisely controlled by visible and UV light irradiations.

The Nyquist plots shown in Figure 5d confirm that the conductivity is highly related to the concentrations of [AzoC_6_MIM][TFSI]. Additionally, the impedance varies with concentrations, and the capacitive characteristics also change with the different compositions of the fabrics. Because of the high dielectric constant of PVDF-HFP, its high polarization properties can contribute to the capacitive characteristics of the fabrics. The fabrics with a higher concentration of PVDF-HFP result in an increased imaginary impedance (−Z′′) in the low-frequency region because the changes in this region are slower and easier to polarize, which leads to a more pronounced capacitive behavior. In Figure 5e, it is observed that the conductivity of the fabric with 20 wt % [AzoC_6_MIM][TFSI] is slightly higher than that of the fabric with 10 wt % [AzoC_6_MIM][TFSI]. Interestingly, the fabric with 30 wt % [AzoC_6_MIM][TFSI] is much more conductive than fabrics with 10 or 20 wt % [AzoC_6_MIM][TFSI] regardless of *trans* or *cis* forms. It is hypothesized that fusion occurs in the fabric with excessively high concentrations of [AzoC_6_MIM][TFSI]. The phenomenon enhances the mobilities of the ionic liquids, which leads to better conductivities.

Furthermore, the conductivities of a posthealed fabric are also demonstrated in the time-resolved impedance test, as shown in Figure 5f. After the impedance of the fabric is stabilized, the fabric is cut in the middle with a blade. The damage causes a rapid increase in impedance that is nearly equivalent to an open circuit condition. The cut pieces of fabrics are then overlapped and pressed with binder clips. In the experiments, we observed that the overlap of the cut fabrics can affect the impedance with decreasing values. Therefore, two pieces of fabrics are approached with minimum overlapping regions to avoid a drastic decrease of impedance because of thickening the fabrics. After healing of the overlapped pieces of fabrics, the impedance nearly recovers to its undamaged state. Still, the value of impedance is slightly lower than that of the original one because of the doubled thickness at the healing part caused by overlapping. Moreover, after the fabric is self-healed in approximately 10 min, the impedance further decreases and stabilizes. The results demonstrate that the recoverability of the fabric can be achieved after the healing process.

## Conclusions

In this work, we introduce healable and photoresponsive electrospun fabrics composed of PVDF-HFP, TPU, and [AzoC_6_MIM][TFSI]. The resulting nonwoven fabrics exhibit stability, elasticity, water resistance, and photoresponsiveness to UV and visible lights. When PVDF-HFP is solely blended with [AzoC_6_MIM][TFSI] for the electrospinning process, a film-like, nonporous structure forms because of intense ion-dipole interactions. The incorporation of TPU prevents the fibrous structure from fusing. Additionally, TPU enhances the mechanical properties of the fabrics such as stretchability and tensile stress. The ion-dipole interactions between PVDF-HFP and [AzoC_6_MIM][TFSI] endow the fabrics with an intrinsic self-healing ability. At optimal concentration, the posthealed fabrics can restore up to 97% of their original mechanical properties, as demonstrated in the tensile test. Due to the presence of azobenzene-based ionic liquids, the fabrics also exhibit reversible responsiveness with controlled electrical properties when irradiated by UV and visible lights. The demonstrations of the multifunctional fabrics with integrated ionic liquids open new possibilities for applications in fabric-based wearable devices.

## Experimental Section

### Materials

Poly(vinylidene fluoride-*co*-hexafluoropropylene) (PVDF-HFP) was obtained from 3 M Dyneon Fluoroelastomer FE. Thermoplastic polyurethane (TPU, 1685A-E2) was purchased from Great Eastern Resins Industrial Corporation (GRECO, Taiwan). 1-Methylimidazole (99%), lithium bis(trifluoromethanesulfonyl)imide (LiTFSI), sodium hydroxide (100%), and acetonitrile (ACN, anhydrous, 99.8%) were purchased from Sigma-Aldrich. Acetone (99%), isopropanol (99.5%), ethanol (99.5%), tetrahydrofuran (THF, 99.5%), and *N*,*N*-dimethylformamide (DMF, 99.8%) were obtained from Echo Chemical. Aniline (>99%) was purchased from Alfa Aesar. Sodium nitrite (98%) and potassium carbonate (99%) were bought from Duksan. 1,6-Dibromohexane (98%) and phenol (99%) were obtained from Acros Organics. [AzoC_6_MIM][Br] was synthesized according to the previous study.^27^

### Synthesis of [AzoC_6_MIM][TFSI]

The complete synthetic route of[AzoC_6_MIM][TFSI] is shown in Figure 2a. [AzoC_6_MIM][TFSI] was obtained by an anion exchange reaction of [AzoC_6_MIM][Br]. First, [AzoC_6_MIM][Br] was dissolved (0.37 g, 1 mmol) in DI water (100 mL) in a round-bottom flask. Lithium bis(trifluoromethanesulfonyl)imide (0.29 g, 1 mmol) was then added dropwise. Subsequently, the mixture was stirred for 1 h at 25 °C. Yellow precipitates were obtained after the filtration, followed by washing three times with DI water. Finally, [AzoC_6_MIM][TFSI] was received by drying at 40 °C for 24 h in vacuum. ^1^H NMR (400 MHz, chloroform-d, δ ppm): 9.59 (s, 1H), 7.89 (d, *J* = 14.5 Hz, 4H), 7.52–7.48 (m, 2H), 7.44 (d, *J* = 7.1 Hz, 1H), 7.19 (s, 2H), 6.99 (d, *J* = 9.0 Hz, 2H), 4.28 (t, *J* = 7.6 Hz, 2H), 4.05 (t, *J* = 6.2 Hz, 2H), 4.01 (s, 3H), 2.00–1.92 (m, 2H), 1.87–1.80 (m, 2H), 1.78–1.72 (m, 2H), 1.49–1.42 (m, 2H).

### Fabrication of [AzoC_6_MIM][TFSI]/PVDF-HFP/TPU Electrospun Fabrics

First, PVDF-HFP, TPU, and [AzoC_6_MIM][TFSI] were mixed to form the polymer matrix, including 10, 20, or 30 wt % of [AzoC_6_MIM][TFSI]. The proportions of PVDF-HFP and TPU in the matrix are 90, 80, or 70 wt %, respectively, and the ratio of PVDF-HFP and TPU was kept 5:4. The polymer matrix was then dissolved in a solvent mixture of DMF and THF (1:1 w/w) to prepare a 25 wt % polymer solution. Subsequently, the solution was stirred at 200 rpm for 8 h at room temperature to form a well-dispersed solution. After that, the 3 mL well-dispersed solution was filled in a plastic syringe. A needle with a 22 gauge (Hamilton, blunt head, inner diameter: 0.41 mm) was then connected to the syringe. The distance between the needle and the collecting plate, which was wrapped with aluminum foil, was maintained as 14 cm. The flow rate was controlled by a syringe pump (KD Scientific) and was kept at 1 mL/h. The power supply (SIMCO) was connected to the needle to apply a voltage of 20 kV for the electrospinning process. After fibers were collected on the aluminum foil for ∼4 h, electrospun fabrics were obtained, followed by drying under ambient conditions for 24 h.

### Mechanical and Healing Test

The mechanical properties and cyclic tests were measured by using a tensile testing machine (Shimadzu, EZ). The fabrics were cut into 1 cm × 3 cm, followed by fixing on 3 cm × 8 cm plates with 1 cm × 2 cm testing areas for stretching. In the healing process, a 1 cm × 3.5 cm fabric was cut in the middle, and the damaged region was then overlapped by 0.5 cm. The overlapped regions were sandwiched between two glass slides and pressed with a weight of 1 kg for 12 h. The tensile test was conducted at a strain rate of 4 mm/min. The strains (%) were compared between the undamaged and the posthealed fabrics to evaluate the healing abilities.

### Electrical Property Test

Electrical properties were measured by electrochemical impedance spectroscopy (EIS) (CHI 6116E). A fabric was sandwiched between two ITO glasses. The edges of the ITO glasses were connected to EIS by using copper foils. Measurements were carried out under two conditions: UV and visible light irradiations. The frequency was set from 10,000 to 10 Hz at 0.5 V to obtain the Nyquist plots. Before the measurements started, both conditions were stabilized for more than 1 h. The data of the reversibility test were obtained from a time-resolved impedance test at 1000 Hz and 0.5 V. The samples were alternately irradiated with UV light for 5 min and visible light for 30 min.

### Analysis and Characterization

The morphologies of the samples were observed by using a scanning electron microscope (SEM, JEOL, JSM-7401F) at the micrometer scale. For SEM measurements, samples were dried by using a vacuum pump for 4 h and then coated with approximately 4 nm of platinum by using a JEOL JFC-1600 sputter coater at 20 mA for 50 s to enhance conductivity. Atomic force microscopy (AFM, Innova) with a soft tapping mode was also used for determining the surface morphologies of the fiber structures and heights. UV–vis reflection spectra of fabrics (20 wt % [AzoC_6_MIM][TFSI]) were measured from 350 to 700 nm using a Hitachi U-4100 spectrometer. UV–vis absorption spectra of a [AzoC_6_MIM][TFSI] solution (0.2 mM in ethanol) were measured from 250 to 600 nm using a U-2900 spectrometer. Fourier-transform infrared (FTIR) analyses were conducted using an FTIR spectrometer (PerkinElmer Spectrum One) to analyze the fabrics. Differential scanning calorimetry (DSC, TA Q200) measurements were performed from −80 to 100 °C with a heating ramp of 10 °C/min for samples of 1 mg. ^1^H NMR spectra were recorded with a NMR spectrometer (JEOL, ECZ500R/S1). Thermogravimetric analysis (TGA, TA55) was conducted from 50 to 700 °C for samples of 5 mg.

## Supporting Information Available

^1^H NMR and ^19^F NMR spectra of [AzoC_6_MIM][TFSI]; EDS and SEM images of an [AzoC_6_MIM][TFSI]/PVDF-HFP/TPU fabric; TGA curves and DSC spectra of an [AzoC_6_MIM][TFSI]/PVDF-HFP/TPU fabric and polymers; SEM and AFM images of fabrics with different contents; stress–strain curves of fabrics with different concentrations of TPU; cyclic tensile test data of fabrics with different concentrations of [AzoC_6_MIM][TFSI] (PDF)
