# Supplementary material for: Intrinsically Healable and Photoresponsive Electrospun Fabrics: Integrating PVDF-HFP, TPU, and Azobenzene Ionic Liquids
Source: ACS Appl Mater Interfaces. 2024 Dec 23;17(1):2215–23. doi: 10.1021/acsami.4c17199 (PMC11783359; doi:10.1021/acsami.4c17199)
Supplement: Supplementary file 1 — am4c17199_si_001.pdf [file am4c17199_si_001.pdf]

Supporting Information

Intrinsically Healable and Photo-Responsive Electrospun Fabrics: Integrating PVDF-HFP, TPU, and Azobenzene Ionic Liquids

Chun-Chi Chang,[1] Lin-Ruei Lee,[1] Sheng Zheng,[1] Tse-Yu Lo,[1] Chia-Wei Chang,[1] Chia-Ti Wu,[1] Tsung-Hung Tsai,[1] Huan-Ru Chen,[1] Yi-Fan Chen,[1] Ming-Hsuan Chang,[1] and Jiun-Tai Chen[1,2]*

[1]Department of Applied Chemistry, National Yang Ming Chiao Tung University, Hsinchu, Taiwan 300093

[2]Center for Emergent Functional Matter Science, National Yang Ming Chiao Tung University, Hsinchu, Taiwan 300093

*To whom correspondence should be addressed. E-mail: jtchen@mail.nctu.edu.tw. Tel.: +886-3-5731631

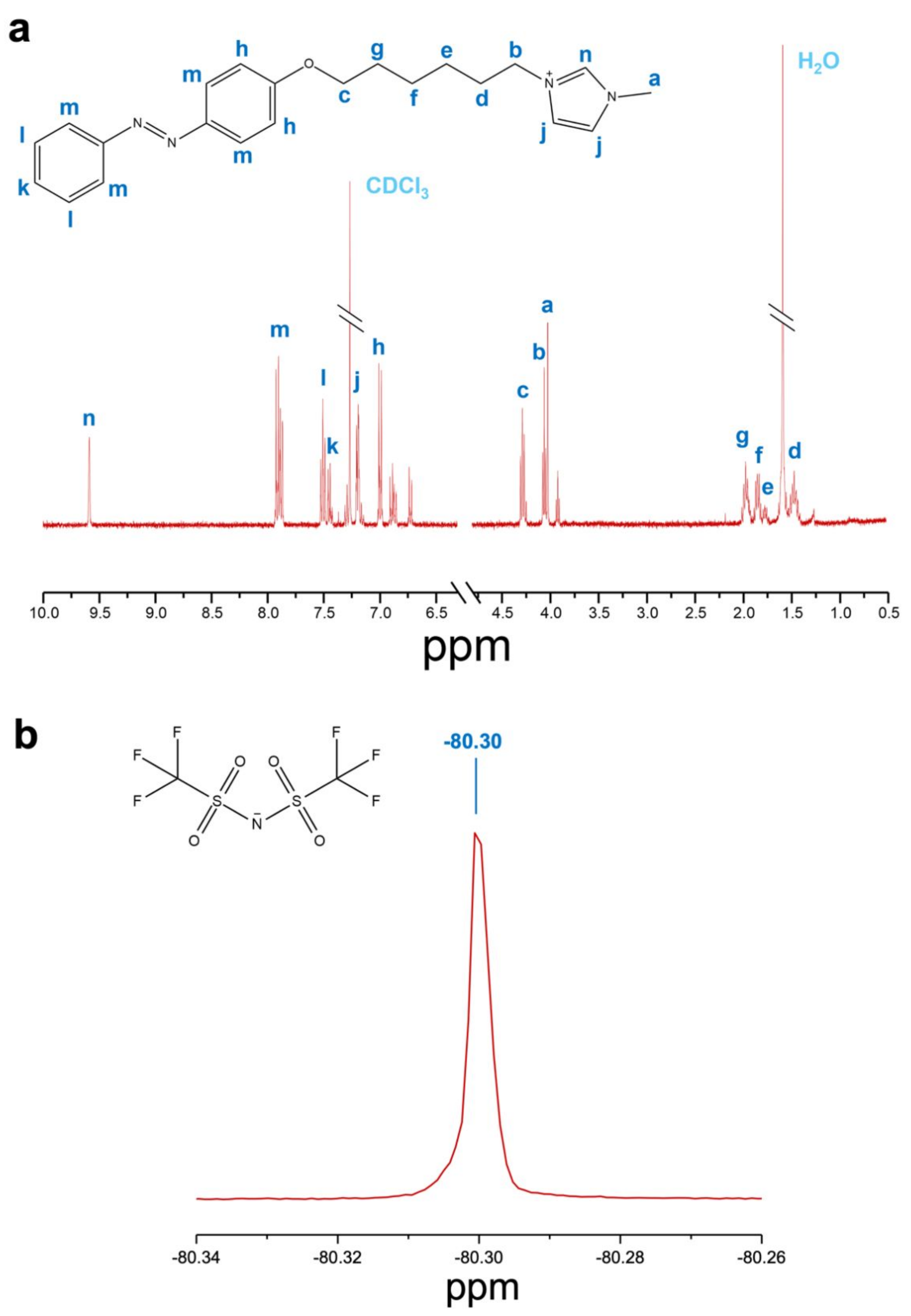

Figure S1. (a) ^{1}H NMR and (b) ^{19}F NMR spectra of [AzoC_6MIM][TFSI].

Table S1. FTIR peak assignments of [AzoC_6MIM][TFSI]/PVDF-HFP/TPU fabrics

Compound	Wavelength (cm^{-1})	Assignment
PVDF-HFP	840	C-F stretching
	1117	CF_2 antisymmetric stretching
	1392	CH_2 bending
TPU	1530	N-H bending
	1710	C=O stretching
	3320	N-H stretching
[AzoC_6MIM][TFSI]	1310	SO_2 antisymmetric stretching
	1352	N=N stretching

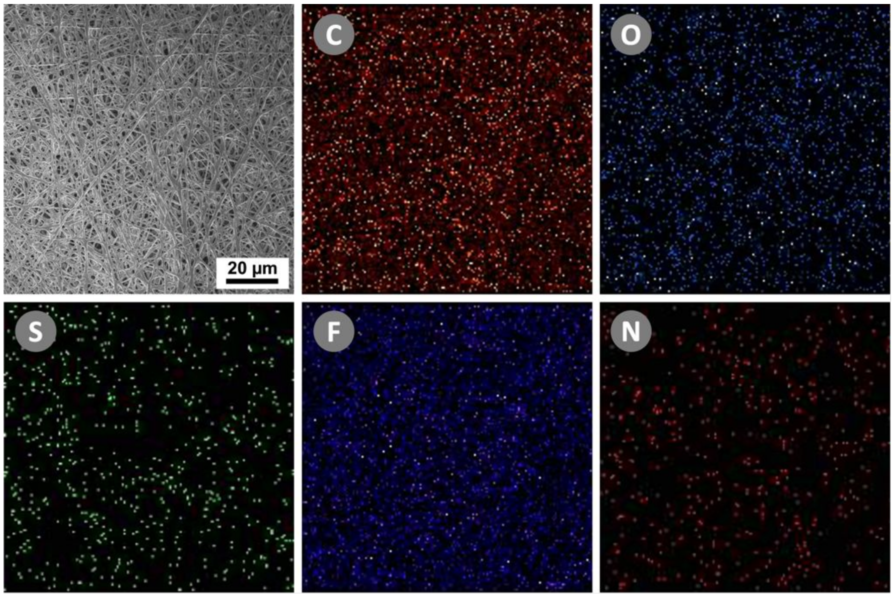

Figure S2. SEM and EDS images of a fabric with 20 wt % [AzoC_6MIM][TFSI].

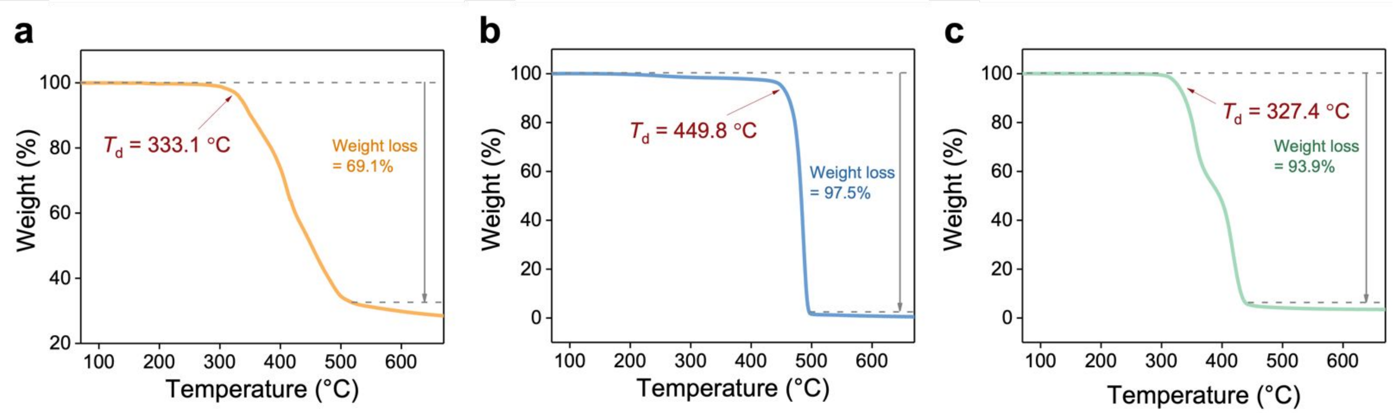

Figure S3. TGA curves of (a) 20 wt % [AzoC$_6$MIM][TFSI]/PVDF-HFP/TPU fabric, (b) neat PVDF-HFP, and (c) neat TPU.

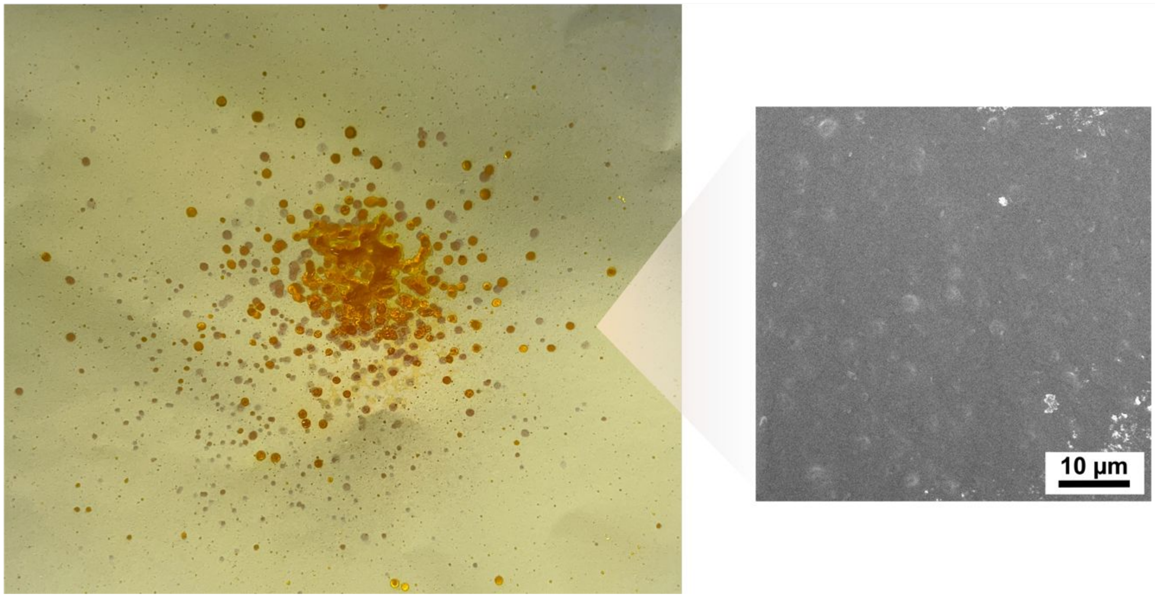

Figure S4. Photo and SEM image of an [AzoC$_6$MIM][TFSI]/PVDF-HFP fabric without TPU (unstable electrospinning jet sprayed).

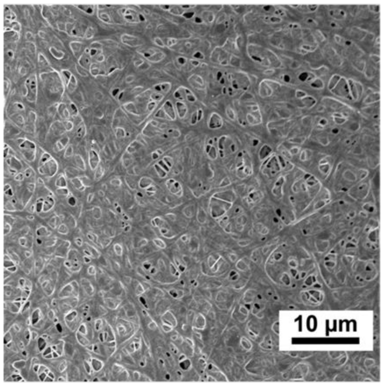

Figure S5. SEM image of a fabric with 35 wt % of [AzoC$_6$MIM][TFSI]. The ratio of PVDF-HFP:TPU=5:4.

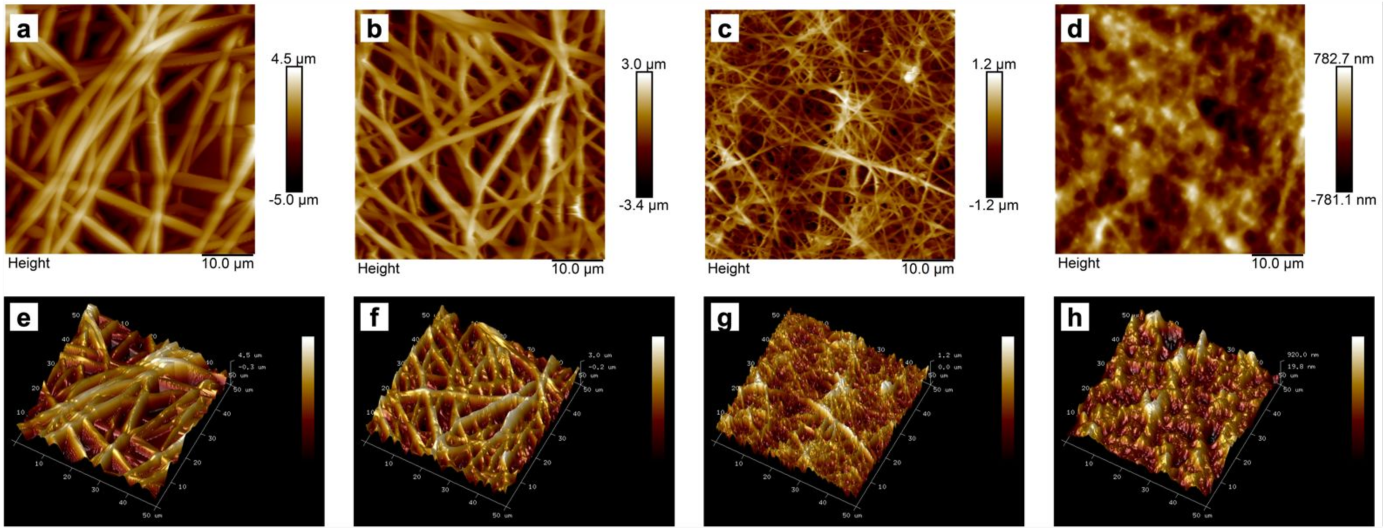

Figure S6. (a–d) AFM 2D height images of fabrics with different contents: (a) 0, (b) 20, and (c) 30 wt % of [AzoC$_6$MIM][TFSI] and (d) without TPU (20 wt % [AzoC$_6$MIM][TFSI]). (e–h) Corresponding AFM 3D images of fabrics shown in (a–d).

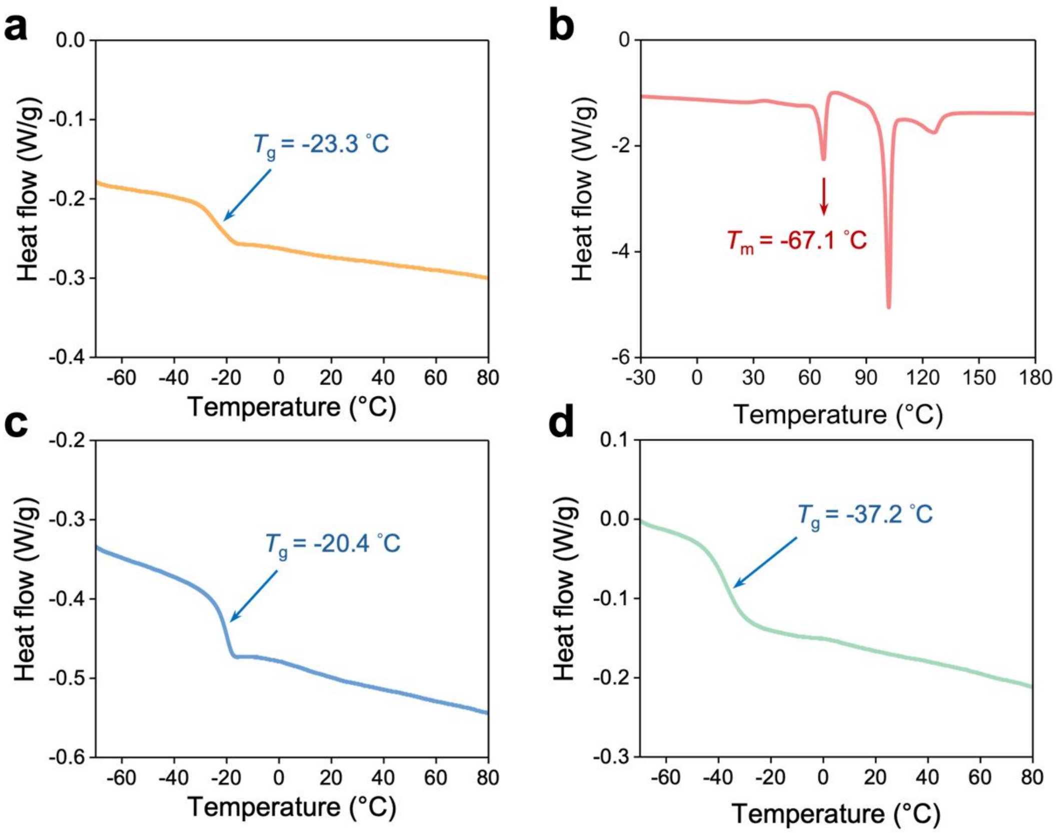

Figure S7. DSC spectra of (a) 20 wt % [AzoC$_6$MIM][TFSI]/PVDF-HFP/TPU fabric, (b) [AzoC$_6$MIM][TFSI] powder, (c) neat PVDF-HFP, and (d) neat TPU.

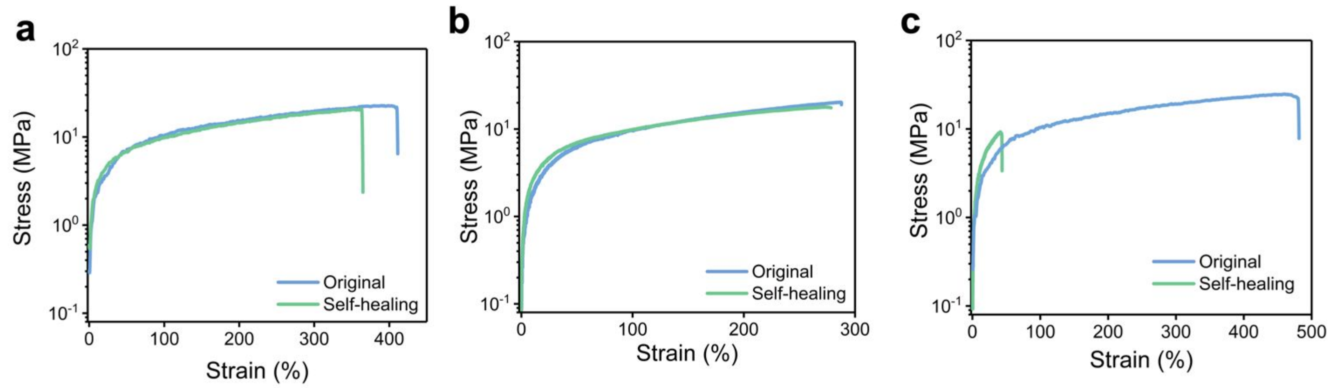

Figure S8. (a-c) Stress-strain curves of fabrics with different concentrations of TPU with fixed concentration of [AzoC$_6$MIM][TFSI] (20 wt %) obtained from tensile test: (a) 20, (b) 35, and (c) 50 wt % of TPU.

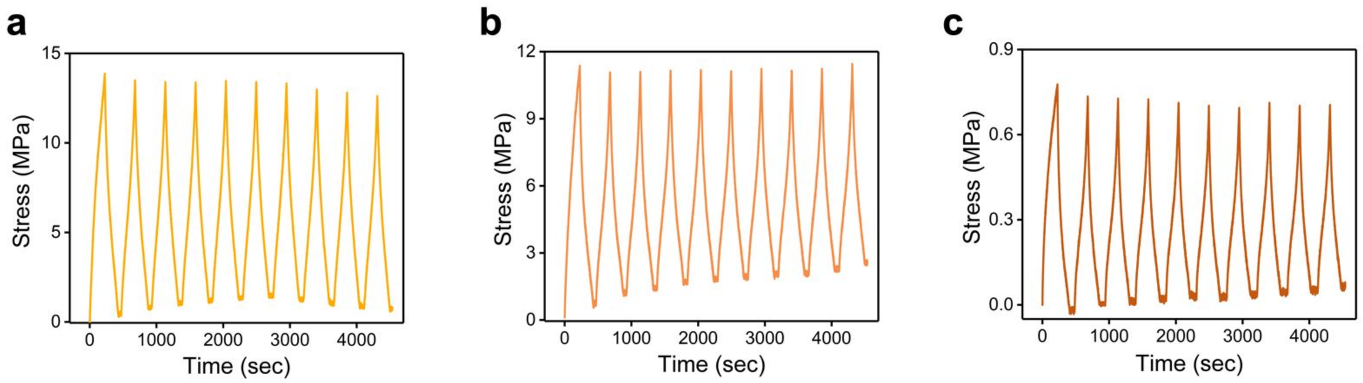

Figure S9. Cyclic tensile tests of fabrics containing (a) 10, (b) 20, and (c) 30 wt % [AzoC_6MIM][TFSI], subjected to 10 cycles of stretching from the original length to a strain of 75%.
